# [^18^F]fluorocholine PET *vs*. [^99m^Tc]sestamibi scintigraphy for detection and localization of hyperfunctioning parathyroid glands in patients with primary hyperparathyroidism: outcomes and resource efficiency

**DOI:** 10.2478/raon-2024-0058

**Published:** 2024-11-28

**Authors:** Sebastijan Rep, Klara Sirca, Ema Macek Lezaic, Katja Zaletel, Marko Hocevar, Luka Lezaic

**Affiliations:** Division of Nuclear Medicine, University Medical Centre Ljubljana, Ljubljana, Slovenia; Medical Imaging and Radiotherapy Department, Faculty of Health Sciences, University of Ljubljana, Ljubljana, Slovenia; Department of Oncological Surgery, Institute of Oncology Ljubljana, Ljubljana, Slovenia; Charité Universitätsmedizin Berlin, Humboldt-Universität zu Berlin, Berlin, Germany; Faculty of Medicine, University of Ljubljana, Ljubljana, Slovenia

**Keywords:** hyperparathyroidism, surgery, parathyroidectomy, cost-effectiveness, [^18^F]choline, [^99m^Tc]sestamibi, PET/CT, SPECT/CT

## Abstract

**Background:**

Minimally invasive parathyroidectomy is the treatment of choice in patients with primary hyperparathyroidism (PHP), but it needs a reliable preoperative localization method to detect hyperfunctioning parathyroid tissue. Higher sensitivity and lower radiation exposure was demonstrated for [^18^F]fluorocholine PET/CT (FCh-PET/CT) in comparison to [^99m^Tc]sestamibi (MIBI) scintigraphy. However, data of its efficiency in resource use and patient outcomes is lacking. The aim of our study was to determine the resource efficiency and patient outcomes of FCh-PET/CT in comparison to conventional MIBI scintigraphy.

**Patients and methods:**

A group of 234 patients who underwent surgery after MIBI scintigraphy was compared to a group of 163 patients who underwent surgery after FCh-PET/CT. The whole working process from the implementation of imaging to the completion of surgical treatment was analyzed. The economic burden was expressed in the time needed for the required procedures.

**Results:**

The time needed to perform imaging was reduced by 83% after FCh-PET/CT in comparison to MIBI scintigraphy. The time needed to perform surgery was reduced by 41% when intraoperative parathyroid hormone monitoring was not used. There was no significant difference in the time of surgery between FCh-PET/CT and MIBI scintigraphy.

**Conclusions:**

FCh-PET/CT reduces the time of imaging, the time of surgery and potentially reduces the number of reoperations for persistent disease.

## Introduction

Primary hyperparathyroidism (PHP) is a condition that develops as a result of increased and uncontrolled production and secretion of parathyroid hormone (PTH) from hyperfunctioning parathyroid glands (HPG). The diagnosis of PHP is typically established through biochemical tests by confirming elevated PTH levels in a patient with hypercalcemia and exclusion of alternative causes.^1^ The therapy of choice in PHP is the surgical removal of the HPG. The traditional surgical approach in primary hyperparathyroidism involves bilateral neck exploration and evaluation of all four parathyroid glands. Due to the development of morphological and functional imaging diagnostics of HPG, a targeted approach has been established in the last decades in which only the area where imaging procedures indicate a solitary HPG is explored through a minimal 2 cm incision (minimally invasive parathyroidectomy, MIP). Accurate preoperative localization is crucial for optimal treatment results using MIP.^2,3^

Imaging procedures are performed only after biochemical confirmation of PHP and are used to plan the operative approach. Parathyroid scintigraphy with [^99m^Tc]sestamibi (MIBI) using several recommended protocols (in particular single-photon emission computed tomography combined with computed tomography, SPECT/CT) in conjunction with neck ultrasound (US) is the reference method for the preoperative localization of HPG.^4,5,6^

In recent years, positron emission tomography/computed tomography (PET/CT) with [^18^F]fluorocholine (FCh-PET/CT) emerged as the most accurate technique in preoperative localization of HPG.^7^ Results from both early and later studies have shown that the sensitivity of FCh-PET/CT is superior to other molecular imaging approaches, including subtraction scintigraphy (SS) and single photon emission computer tomography/computed tomography (SPECT/CT) with MIBI. This is particularly evident in studies that directly compared scintigraphy protocols with FCh-PET/CT as a first-line approach.^8,9,10,11,12,13^ Further advantage of FCh-PET/CT is markedly lower radiation exposure compared to MIBI SS and SPECT/CT.^14^

However, the outcomes and the economic impact of the use of FCh-PET/CT have not been extensively studied. The aim of our study was to compare the clinical outcomes and the resource efficiency of FCh-PET/CT versus MIBI SS combined with SPECT/CT in patients with PHP in the local setting.

## Patients and methods

### Clinical outcomes

This retrospective analysis (approval No. 0120-582/2021/4 by the Committee for Medical ethics of the Republic of Slovenia) covered the period from 2008 to 2016 and included 234 patients who underwent surgery after MIBI SS in combination with SPECT/CT and 163 patients who underwent surgery following FCh-PET/CT. Due to the retrospective nature of the analysis the request for patient consent was waived. We evaluated the success of surgery performed after MIBI SS and SPECT/CT imaging and after FCh-PET/CT imaging and the need for additional surgery in patients with persistent PHP.

### Resource efficiency

As the cost of diagnostic imaging, surgery and hospitalization vary depending on the healthcare environment in which they are performed^15,16^, we evaluated the burden of imaging and surgical procedures by measuring the time required to complete the procedure. This evaluation included the number of imaging procedures per day and/or in the number of hours required by the various occupational groups involved in the process. We assessed the workflow of radiopharmacists, nurses, radiographers (technologists) and physicians. A brief description of the working process for each professional profile is given in Table 1.

**TABLE 1.** Workload of the profiles involved in the imaging process

	**No. workers**	**SS + SPECT (hours)**	**No. patients/day**	**FCh PET (hours)**	**No. patients/day**
**Pharmacist**	1		3 (12)^*^		12
RP preparation		0.7 (2.8)^*^		0,25	
**Nurse**	1				
Cannula placement		0.75 (3)^*^		3	
Cannula removal		0.3 (1.2)^*^		1,2	
**Technologist**	2				
RP application		0.75 (3)^*^		1,2	
Imaging time		6 (24)^*^		4	
QC dally test		0.5 (2)^*^		0,5	
**Physician**	1				
PH&CE and writing a report		3 (12)^*^		12	

* time required for 12 patients to perform SS + SPECT/CT

FCh PET = [^18^F]fluorocholine positron emission tomography; PH&CE = patient history and clinical examination; RP = radiopharmaceutical; QC = quality control; SS + SPECT/CT = subtraction scintigraphy and single-photon emission tomography/computed tomography

### Diagnostic imaging

The SS was performed on a Siemens BasiCAM planar gamma camera at 10 and 90 minutes after the intravenous (i.v.) injection of 600 MBq of MIBI. After the completion of imaging at 90 minutes, the patient was left in the same position and 150 MBq of [^99m^Tc]O_4_^−^ was injected i.v. and identical imaging was performed after 10 minutes. After completion of imaging, a SS was generated by processing where the planar image obtained with [^99m^Tc] O_4_^−^ was subtracted from the planar image obtained with MIBI.

SPECT/CT imaging was performed on a SIEMENS Simbia® T2 gamma camera (Siemens Medical Solutions, Erlangen, Germany), SPECT/CT imaging of the neck and chest was performed 30 minutes after the i.v. injection of 600 MBq of MIBI. Typically, three patients per day were examined. The combination of dual-phase, SS and SPECT/CT is described as “hybrid” imaging protocol.

**FIGURE 1.**
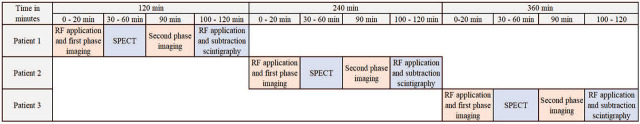
Time workflow/scheme of radiopharmaceutical (RP) application and imaging of subtraction scintigraphy (SS) and single-photon emission tomography/computed tomography (SPECT/CT) in a working day. Approximate times are taken into account for all procedures.

**FIGURE 2.**
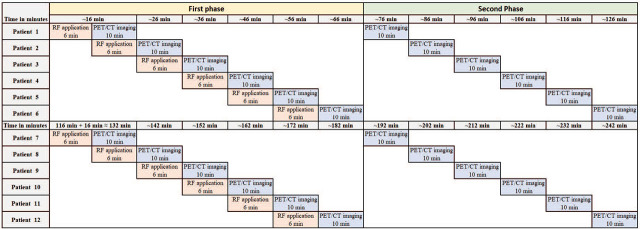
Time workflow/scheme of radiopharmaceutical (RP) application and imaging of [^18^F]fluorocholine positron emission tomography (FCh-PET) in a working day. Approximate times are taken into account for all procedures.

PET imaging was performed on a SIEMENS Biograph mCT® 128 system. PET imaging of the neck and chest was performed 5 and 60 minutes after i.v. injection of 100 MBq of FCh. The vial supplied by the manufacturer contains 2700 MBq FCh in the prescribed volume. The daily delivered activity typically allowed us to examine 12 patients per day, divided into two groups of 6 patients. The daily workflow and occupancy of imaging equipment for both examinations is shown in Figures 1 and 2.

### Surgery

The time of the surgical procedure was recorded from the arrival of the patient in the operating room to the departure from it.

In patients with a solitary HPG on MIBI SS and SPECT/CT or FCh-PET/CT only a focused (lateral trapdoor approach) parathyroidectomy without intraoperative PTH (ioPTH) testing was performed. In patients with multiple lesions or nonlocalization a more extensive classical bilateral neck exploration with ioPTH testing was performed. PTH measurements (in pg/mL) were performed at the induction of general anesthesia and 10 min after the removal of the last enlarged parathyroid gland and the procedure was deemed successful if there was a ≥ 50% drop from baseline. For determination of ioPTH, blood samples were collected into K2-EDTA Vacutainer™ tubes (Becton-Dickinson, Plymouth, UK), centrifuged (2,500 g, 10 min, room temperature) and analysed using a commercially available kit (cobas PTH STAT) and analyzer (cobas e 411; both Roche Diagnostics, Mannheim, Germany).

Patients were discharged from the hospital on the next day and in all patient’s serum values of Ca^2+^ and PTH were obtained on the morning before discharge. Normalization of Ca^2+^ and PTH values was considered to represent successful surgery.

### Statistical analysis

Differences in the required time to completion were expressed as percentages. The Kolmogorov-Smirnov test was used for the evaluation of the normality of data distribution and Mann-Whitney test to confirm the difference between the independent variables. A p < 0.05 was considered statistically significant.

## Results

### Clinical outcomes

For the evaluation of the surgical part, we reviewed the 4-year period before and after the implementation of FCh-PET/CT (2008 to 2016) for preoperative localization of HPG. The period before the implementation included 234 patients and the period after the implementation included 163 patients. All localized HPG were surgically removed. Persistent hypercalcemia was more often observed in patients who underwent MIBI SS and SPECT/CT prior to surgery (15.8%) in comparison to those who underwent FCh-PET/CT (2.4%) (p < 0.001). Reoperation was required in 12.3% of patients who underwent SS and SPECT/CT prior to the initial surgery, compared to 1.8% of patients who had FCh-PET/CT. Postoperative complications occurred in 5.3% of patients after SS and SPECT/CT and 4.7% of patients after FCh-PET/CT. Detailed data are presented in Table 2.

**TABLE 2.** The most common causes (percentages) of postoperative complications

	**SS and SPECT/CT**	**FCh PET**
Tingling	4/234 (1.7%)	3/163 (1.8%)
Chvostek sign	1/234 (0.4%)	1/163 (0.6%)
Hungry bone syndrome	1/234 (0.4%)	2/163 (1.2%)
Malaise	1/234 (0.4%)	N/C
Hoarseness	1/234 (0.4%)	N/C
Postoperative Hypocalcemia	1/234 (0.4%)	N/C
Hematoma	1/234 (0.4%)	N/C
Deterioration of renal function	N/C	1/163 (0.6%)
Reoperation	29/234 (12.3%)^*^	3/163 (1.8%)^*^
**All**	39/234 (16.6%)^**^	10/163 (6.1%)^**^

* p < 0.001 for SS and SPECT/CT *vs.* FCh PET;

** p < 0.001 for SS and SPECT/CT *vs.* FCh PET

FCh PET = [^18^F]fluorocholine positron emission tomography; N/C = no case; SS and SPECT/CT = subtraction scintigraphy and single-photon emission tomography/computed tomography

### Resource efficiency

The time required for surgery based on the results of MIBI SS and SPECT/CT versus FCh-PET/CT was comparable (Table 3). However, the implementation of ioPTH monitoring significantly prolonged the time of surgery, while the duration of hospitalization did not differ significantly (Table 4).

**TABLE 3.** The patient number and the required time of surgery expressed in minutes after subtraction scintigraphy (SS) and single-photon emission computed tomography/computed tomography (SPECT/CT) *vs.* [^18^F]fluorocholine positron emission tomography (FCh-PET)

	**All pts**	**Mean**	**Median**	**SD**	**Min**	**Max**
MIBI SS and SPECT/CT	234	67.37	60.00	36.88	20.00	280.00
FCh-PET	163	70.79	55.00	38.21	25.00	195.00
p		0.66				
	**Solitary HPG**	**Mean**	**Median**	**SD**	**Min**	**Max**
MIBI SS and SPECT/CT	195	63.56	50.00	33.04	20.00	235.00
FCh-PET	138	64.42	50.00	33.04	25.00	180.00
p		0.93				
	**Multiple HPG**	**Mean**	**Median**	**SD**	**Min**	**Max**
MIBI SS and SPECT/CT	39	94.26	100.00	43.65	25.00	235.00
FCh-PET	25	104.79	107.00	40.79	30.00	180.00
p		0.23				

HPG = hyperfunctioning parathyroid gland(s); Max = maximum; Min = minimum; pts = patients; SD = standard deviation

**TABLE 4.** Influence of probable prognostic factors on overall survival (OS) and deasise-free survival (DFS)

	**All pts**	**Mean**	**Median**	**SD**	**Min**	**Max**
No ioPTH	123	60.00	50.00	33.91	25.00	195.00
ioPTH	39	103.00	100.00	31.27	40.00	195.00
p		< 0.001				
	**Solitary HPG**	**Mean**	**Median**	**SD**	**Min**	**Max**
No ioPTH	113	57.30	50.00	31.05	25.00	180.00
ioPTH	25	96.60	90.00	29.71	40.00	155.00
p		< 0.001				
	**Multiple HPG**	**Mean**	**Median**	**SD**	**Min**	**Max**
No ioPTH	10	85.55	70.00	49.90	30.00	195.00
ioPTH	15	116.33	110.00	30.49	80.00	195.00
p		0.03				

HPG = hyperfunctioning parathyroid gland(s); ioPTH = intraoperative parathyroid hormone determination; Max = maximum; Min = minimum; pts = patients; SD = standard deviation

Diagrams 1 and 2 (Figures 1 and 2) depict the workflow of the imaging process for SS and SPECT/CT and FCh-PET/CT, respectively. With MIBI SS and SPECT/CT imaging, up to three patients were typically imaged in a day, whereas up to 12 patients were imaged per day with FCh-PET/CT. Assuming a standard imaging workload of 12 patients, pure imaging time was reduced by 83%, radipharmaceutical (RP) preparation time by 89% and quality control (QC) procedures by 75% with the use of FCh-PET/CT (as derived from Table 1). There were no differences in the time required for cannula placement and removal, RP injection/application, the time needed for patient history and clinical examination (PH&CE), and for writing the report.

## Discussion

Parathyroid scintigraphy for the preoperative localization of HPG in patients with PHP enabled the introduction of MIP as the optimal surgical approach in patients with solitary HPG (approximately 85% of all patients with PHP). The main advantages of MIP include the possibility of using local/regional anaesthesia, shorter operative time, better cosmetic results and a more favourable extent of initial surgery in patients requiring repeated surgical treatment due to persistent or recurrent disease.^15,16,17,18,19^ A prerequisite for optimal treatment results using MIP is accurate preoperative localization.^3^

In recent years, various publications have described using FCh-PET/CT in the preoperative localization of HPG in patients with PHP. FCh-PET/CT has been shown to enhance the sensitivity for the localization of HPG in comparison to MIBI SS and SPECT/CT, especially in patients with multi-glandular disease.^8, 10,11,12,13^

Despite excellent diagnostic performance as shown in meta-analyses^7,20^ and lower radiation exposure^14^ of FCh-PET/CT in comparison to conventional MIBI SS and SPECT/CT, the main drawback is the higher cost of the radiopharmaceutical compared to MIBI. Since the price of the radiopharmaceutical is only one aspect of the entire procedure, we evaluated and compared the workflow burden of FCh-PET/CT *vs.* MIBI SS combined with SPECT/CT expressed in the required time for different procedures from the implementation of imaging to the completion of surgical treatment.

The main difference was found in the number of imaging procedures that can be performed in a typical imaging day. While we were able to complete FCh-PET/CT imaging in 12 patients, we were able to complete MIBI SS and SPECT/CT in three patients, resulting in overall four-fold gain for FCh-PET/CT. The main reason is the short imaging time and imaging protocol for FCh PET/CT that allows overlap between patients (Figure 2). All other aspects of the imaging procedure (RP preparation, placement and removal of the cannula, RP injection, PH&CE and writing the report) differed significantly less, if at all, in terms of time requirements. The first reason for the shorter imaging time using PET *vs.* MIBI SS and SPECT/CT is significantly higher sensitivity of the PET detector system in comparison to conventional gamma cameras.^21^ The time requirement for a single phase of FCh-PET/CT imaging – the acquisition time (5 min) and the time needed for the patient to occupy and leave the examination table (5 min) – amounted to 10 minutes. Our routine dual-phase workflow allowed us to image 12 patients in a working day. In contrast, about 2 hours were required per patient to perform MIBI SS and SPECT/CT using the routine hybrid imaging protocol, limiting the number of patients to 3 daily; cumulatively, for a standard 12-patient workload higher burden was found for pure imaging time, RP preparation and QC procedures.

Alternative imaging protocols requiring substantially shorter imaging times are also in routine use.^6^ Subtraction scintigraphy (including tomographic subtraction) can also be performed with Na[^123^I]I, allowing for simultaneous imaging with MIBI due to differing energy windows. this approach can substantially reduce cumulative imaging times, potentially making them comparable to FCh PET, while single-RP protocols using solely dual-phase MIBI SPECT/CT further simplify the procedure; both approaches were shown to result in favourable clinical accuracy.^5^ However, in our experience^8,12^ and that of other groups^22^, the hybrid protocol results in the optimal diagnostic performance when using conventional scintigraphic approaches. Nevertheless, FCh-PET/CT was unequivocally found to be diagnostically superior to conventional imaging.^7^ In summary, FCh-PET/CT offers shorter acquisition times in comparison to MIBI SS and SPECT/CT.^6,23,24,25^ Small additional gains may result from fewer required quality control procedures and reduced reporting times due to the superior image quality of FCh-PET/CT.

Further potential aspect of improved resource efficiency is the superior diagnostic accuracy of FCh-PET/CT which enables MIP without the need for ioPTH. As demonstrated by our group, ioPTH can be safely omitted in patients with solitary HPG on FCh-PET/CT, leading to the reduction of surgery time.^3^ Our results suggest that the average time of surgery can be shortened by as much as 41% if ioPTH monitoring is not performed, which reduces the overall cost of the procedure. In the present analysis the difference in time requirement of the surgical procedure with and without ioPTH was evaluated only in patients operated on the basis of FCh-PET/CT imaging as the method of ioPTH determination was introduced in our institution(s) in 2012 (approximately the time of introduction of FCh-PET/CT). This was reflected in inferior results of MIP in patients operated based on MIBI SS and SPECT/CT imaging without ioPTH assessment in comparison to patients after FCh-PET/CT (reoperation for persistent PHP was required in 12.3% of patients after MIBI SS and SPECT/CT imaging in comparison to only 1.8% of surgeries after FCh-PET/CT imaging). With the use of ioPTH assessment in patients operated after MIBI SS and SPECT/CT imaging the number of reoperations for persistent PHP would probably be lower. However, in a large series comparing the diagnostic accuracy of MIBI SS and SPECT/CT to FCh-PET/CT imaging in pHPT reported by our group, 47% of patients with multiglandular disease on FCh-PET/CT would undergo resection of a single gland detected of conventional scintigraphic imaging^12^; in several of those patients, removal of the largest offending gland would have resulted in the reduction of ioPTH levels above 50% and premature termination of surgery, an occurrence also reported by other groups.^26^

The superior diagnostic performance of FCh-PET/CT over conventional scintigraphic imaging resulted in the recommendation for the method in the current EANM guidelines on parathyroid imaging as an “alternative” first-line imaging approach to be used whenever possible, with the caveat of unclear cost-effectiveness.^6^ Most recently, the superior diagnostic performance of FCh-PET/CT over MIBI SS and SPECT/CT already reported in direct comparison was confirmed in a randomized trial comparing the two methods as a first-line imaging approach: FCh-PET/CT was shown to be superior and safe imaging option.^27^ As the social cost of the compared first-line imaging approaches was part of the study protocol^28^, the additional results are awaited. Indeed, the main barrier for the introduction of FCh-PET/CT into routine clinical practice – in particular as a first-line imaging choice – is the limited availability of the method in many clinical environments, related to locally specific factors such as lack of equipment, prohibitive cost and/or legal limitations of off-label use of the radiopharmaceutical. These limitations are clearly recognized in detailed reviews, meta-analyses and existing guidelines, stating that a cost-effectiveness analysis of the method would be required if not crucial to promote its widespread use.^6–7,20^ Few attempts to assess the cost-effectiveness of FCh-PET/CT in comparison to conventional scintigraphic and alternative radiological imaging methods reflect the significant local differences in availability and reimbursement strategies. In the United States, FCh-PET/CT was found to be a potentially economically viable imaging approach, but expensive with a narrow cost-effectiveness margin.^29^ Conversely, in the EU setting, FCh-PET/CT was shown to be an effective sole, first-line imaging choice with negligible additional expenses.^30^ With clearly superior diagnostic performance and comparable cost, FCh-PET/CT is expected to be promoted as a first-line imaging method of choice in primary hyperparathyroidism.

## Conclusions

FCh-PET/CT reduces the time of imaging, the time of surgery and potentially reduces the number of reoperations for persistent disease. All these advantages may translate into lower overall costs of management of patients with pHP by using FCh-PET/CT in comparison to MIBI SS and SPECT/CT, confirming its appropriate role as a first-line imaging choice.
